# Hybrid diffuse optical appraisal of peripheral and cerebral changes in critically ill patients receiving red blood cell transfusion

**DOI:** 10.1117/1.BIOS.2.1.015001

**Published:** 2025-01-23

**Authors:** Susanna Tagliabue, Anna Rey-Perez, Lourdes Exposito, Andrés F. Jimenez, Sara Valles Angulo, Federica Maruccia, Jonas B. Fischer, Michal Kacprzak, Maria A. Poca, Turgut Durduran

**Affiliations:** a ICFO—Institut de Ciències Fotòniques, The Barcelona Institute of Science and Technology, Barcelona, Spain; b Vall d’Hebron University Hospital, Neurotrauma Intensive Care Unit, Barcelona, Spain; c Vall d’Hebron Research Institute (VHIR), Neurotraumatology and Neurosurgery Research Unit (UNINN), Barcelona, Spain; d Polish Academy of Sciences, Nalecz Institute of Biocybernetics and Biomedical Engineering, Warsaw, Poland; e Vall d’Hebron University Hospital, Department of Neurosurgery, Barcelona, Spain; f Universitat Autónoma de Barcelona, Barcelona, Spain; g Institució Catalana de Recerca i Estudis Avançats (ICREA), Barcelona, Spain

**Keywords:** red blood cell transfusion, anemia, hybrid diffuse optics, critical care

## Abstract

**Significance**: Red blood cell transfusions (RBCTs) to restore hemoglobin concentration and hematocrit values in anemic patients are debated in critical illness.

**Aim**: We used non-invasive hybrid diffuse optics (DO) to study RBCT’s effect on the brain and peripheral muscle evaluated in critically ill patients.

**Approach**: Critically ill patients admitted to the neurotrauma intensive care unit of the Vall d’Hebron University Hospital that required blood transfusion were included in the study and continuously monitored by DO methods alongside time-specific blood gas samples.

**Results**: In 14 patients, blood-derived hemoglobin, hematocrit, and DO-derived metabolic rate of oxygen, oxy- and total hemoglobin significantly increased after RBCT. Oxygen extraction fraction significantly decreased. Blood flow significantly increased only in muscles. Deoxy-hemoglobin concentration remained unchanged. No significant differences were found between cerebral and peripheral signals post-RBCT, except for the metabolic rate of oxygen (higher in muscle).

**Conclusion**: Hybrid DO monitoring quantifies cerebral and peripheral microvascular changes during red blood cell transfusion, enabling insights into oxygen metabolism and advancing critical care management.

Statement of Discovery This study introduces a novel approach to monitoring critically ill patients undergoing red blood cell transfusion by employing non-invasive hybrid diffuse optical monitoring, enabling continuous, real-time assessment of microvascular blood oxygenation, metabolic rate of oxygen and blood flow, parameters that are not usually accessible in this population. The research pioneers the application of this method to characterize and quantify the physiological changes induced by red blood cell transfusion therapy in both cerebral and peripheral tissues, providing a better understanding of the impact of this therapy, which remains controversial in critically injured patients. By demonstrating the feasibility of this approach, this study lays the groundwork for personalized management of the therapy, with the potential to significantly improve patient outcomes by reducing complications such as hypoxia and ischemia.

## 1 Introduction

Anemia is a common complication observed in critically ill patients admitted to the intensive care unit (ICU). In the ICU setting, anemia can result from severe bleeding after trauma, acute blood loss, or hemorrhage during surgery. In addition, it arises in non-bleeding but critically ill patients experiencing a rapid decrease in blood arterial total hemoglobin concentration (Hgb)^1^ (details about the terminology and acronyms used in this article in relation to hemoglobin concentration are reported in Appendix A). Typically, anemia is defined by low arterial oxy-hemoglobin concentration (${\mathrm{HgbO}}_{2}$) in blood or low hematocrit (HCT) percentage (∼8 to 10 g/dL^2^^,^^3^ and 30%,^4^ respectively) relative to normal values (∼14 to 15 g/dL and 35% to 50%, respectively). This is quite common in the general ICU critically ill population within the first three days after admission.

Under anemic conditions, low ${\mathrm{HgbO}}_{2}$ translates into a reduced supply, release, and diffusion of oxygen into tissues. This affects the blood flow [BF, cerebral blood flow CBF when related to the brain, and peripheral blood flow (PBF) when related to peripheral parts of the body]^2^ due to its relationship with oxygen delivery (${\mathrm{DO}}_{2}$), which is the product of BF and arterial oxygen content (${\mathrm{CaO}}_{2}$).^4^^,^^5^ As such, anemia can have deleterious effects on the patient’s health;^2^^,^^5^ thus, severe anemia must be avoided by action taken as early as possible.^6^ Furthermore, patients with severe traumatic brain injury (TBI) usually present low CBF at the beginning of hospitalization,^2^^,^^4^^,^^6^^,^^7^ which increases the risk of ischemia whenever the oxygen supply cannot meet anymore the demand.^2^

Anemic patients generally undergo red blood cell transfusion (RBCT),^1^ with most clinicians accepting this intervention to restore the ${\mathrm{HgbO}}_{2}$ content.^1^^,^^4^ However, RBCT also alters the BF^1^^,^^6^ and other directly related physiological parameters, such as the Hgb, the tissue oxygen saturation (${\mathrm{StO}}_{2}$), the metabolic rate of oxygen (${\mathrm{MRO}}_{2}$), and the oxygen extraction fraction (OEF). In the case of the healthy brain, cerebral autoregulation and other protective mechanisms ensure that these changes do not impair brain function. In patients with severe TBI, however, cerebral autoregulation and the systemic cardiovascular response are frequently compromised.^2^^,^^4^ In non-critical organs, such as peripheral muscles, which lack BF control mechanisms similar to those in the brain, significant and potentially unsustainable alterations may occur. Consequently, the balance between local hemodynamics and metabolism can be disrupted by RBCT.

The indications for RBCT for critical care patients are the subject of considerable debate among clinicians. For example, the “Transfusion Requirements in Critical Care” study, published in 1999^5^^,^^8^ and reviewed in 2012,^9^ questioned the risk-benefit ratio of transfusions and whether they are appropriate in ICU patients with acute brain injuries.^2^^,^^4^^–^^6^^,^^10^ Several studies have reported side effects of RBCT in TBI patients,^4^ and recent guidelines recommend a more restrictive transfusion strategy in these patients, with a ${\mathrm{HgbO}}_{2}$ threshold lower or equal to 7g/dL.^1^^–^^4^^,^^9^^,^^11^^,^^12^ The identification of the hemodynamic and metabolic alterations induced by RBCT in the brain compared with peripheral muscle could offer insights into the specific impact on the vulnerable brain of neurocritical patients.

Our primary argument for this study is that advanced photonic methods utilizing non-invasive hybrid diffuse optics (DO) employing near-infrared light can facilitate a deeper understanding of these phenomena. Hybrid DO, comprising time-resolved spectroscopy (TRS) and diffuse correlation spectroscopy (DCS), could provide a more complete overview of these physiological alterations for the reasons described below.

DCS quantifies the local, microvascular BF (CBF and PBF), whereas TRS quantifies ${\mathrm{StO}}_{2}$, microvascular oxy- and deoxy-hemoglobin concentrations (${[\mathrm{HbO}}_{2}]$ and [Hhb]), total microvascular hemoglobin concentration ([HbT]), and OEF (COEF when cerebral, PEOF when peripheral). Together, they convey information about the ${\mathrm{MRO}}_{2}$ (${\mathrm{CMRO}}_{2}$ when cerebral, ${\mathrm{PMRO}}_{2}$ when peripheral). However, the literature regarding the effects of RBCT on these signals is limited.^13^ Continuous-wave near-infrared spectroscopy (NIRS) is the most commonly used method for measuring blood oxygen saturation^5^^,^^13^ with few studies conducted on children (i.e., premature babies) ^14^^–^^20^ and even fewer on adults.^10^^,^^21^^–^^24^ Hence, further studies such as this one are warranted.

We have conducted a pilot study using hybrid DO for both local cerebral and peripheral continuous monitoring during RBCT in critically ill patients. Therefore, as a first objective, we will validate its usability in such a scenario.

This study was exploratory and observational, analyzing cerebral and peripheral hemodynamic and metabolic biomarkers to elucidate the ability of DO spectroscopic techniques to quantify changes due to RBCT, as our second objective.

### 1.1 Study Hypotheses

Following the stated objectives, our hypothesis posited that RBCT influences DO-derived parameters, prompting us to verify whether we could retrieve significant changes relative to initial baseline values, before RBCT. Analyses were performed at both peripheral and cerebral levels to ascertain potential variations in the response between these sites. This approach allows for the evaluation of potential positive or adverse effects following RBCT, especially putting emphasis on the ability to verify whether any harm is produced to the brain. Therefore, we hypothesized that, although peripheral changes could be seen, RBCT’s effect on the brain should be evaluated to understand it better in this population. This is enabled only by assessing RBCT’s effect on all of the DO-derived parameters at the same time, which is not feasible by standard NIRS monitors.

## 2 Materials and Methods

### 2.1 Study Subjects and Transfusion Criteria

The study obtained clearance from the ethical committee of Vall D’Hebron University Hospital (PR(AG)160/2017) and was conducted following the Declaration of Helsinki.^25^ Written informed consent was obtained before the measurements and signed either by the patient or a legal representative.

Critically ill patients aged 16 to 72 admitted to the neurotrauma ICU of the Vall d’Hebron University Hospital (VHUH) between June 2019 and July 2021 that required blood transfusion were included in the study. Among all critically ill patients, we have recruited: (1) polytrauma patients with [abnormal admission computed tomography (CT) scan] or without (normal CT scan) moderate or severe TBI [Glasgow coma scale (GCS) ≤13], (2) patients with ischemic or hemorrhagic stroke, and (3) patients with severe burns.

As a standard, in this ICU, the anemia threshold was defined as having a ${\mathrm{HgbO}}_{2}\leq 13\;g/\mathrm{dL}$ for adult males and ≤12  g/dL for adult non-pregnant females. To optimize cerebral oxygenation, RBCT was considered in anemic patients. However, blood transfusion was performed in patients with a threshold ${\mathrm{HgbO}}_{2}$ ≤10  g/dL, based on the clinicians’ decision-making, considering the overall patient clinical condition and medical history, and in agreement with the SIBICC. Additional considerations about these thresholds and aspects are reported in Sec. 4.

### 2.2 Study Protocol

**Fig. 1:**
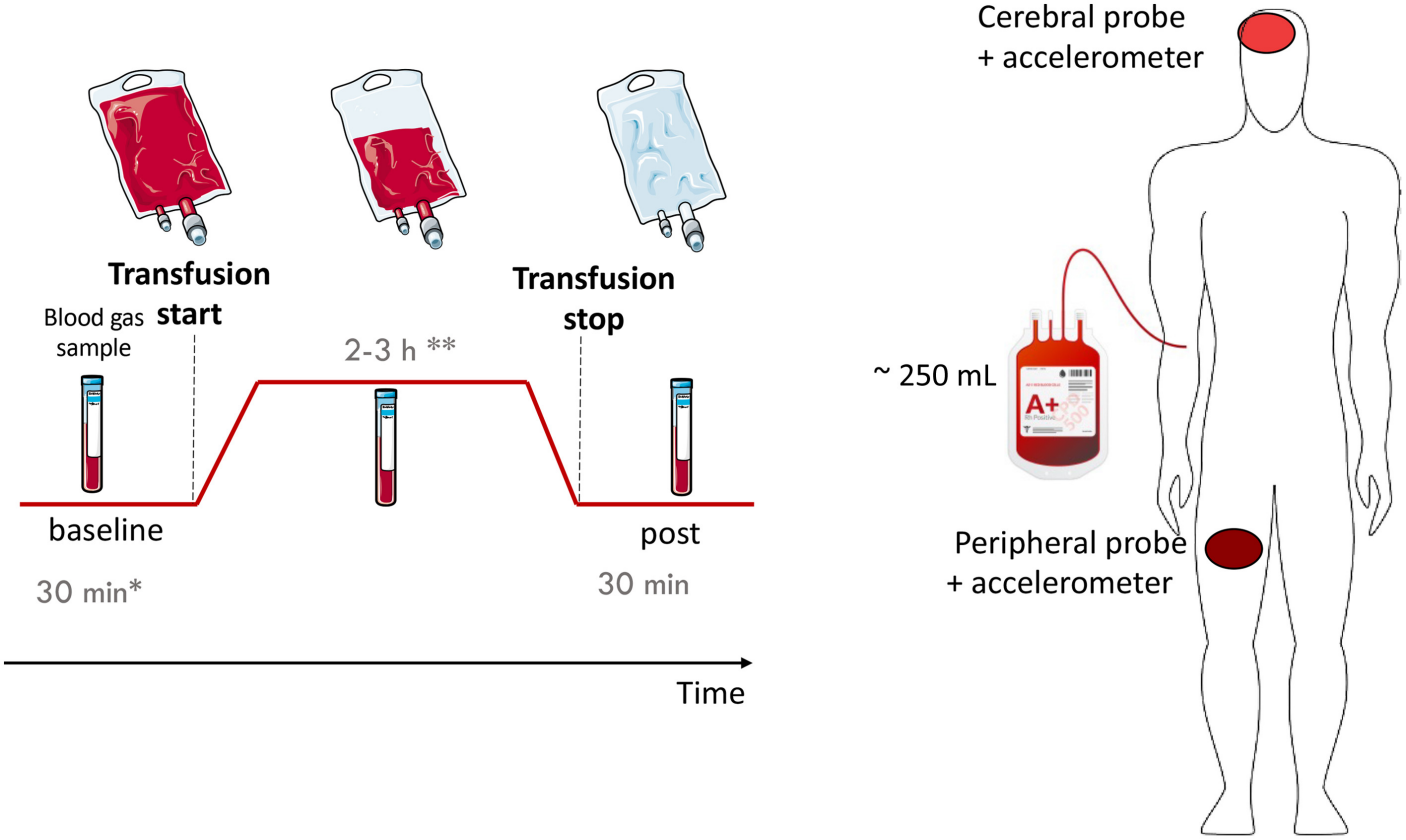
Protocol illustration and optical probe placement. Study protocol: following a baseline pre-RBCT period (*generally ∼30  min but adjusted to the patient’s needs), the RBC transfusion started (**2 to 3 h duration according to the bag volume and/or transfusion rate decided by the nurse) and was measured until finished, when additional 30 min post-transfusion was monitored. Three blood gas samples were taken during each period of the measurement. As depicted on the right, one probe was placed on the forehead of the subject, whereas the second was placed at the peripheral level, generally on the quadriceps femoris. This figure was created by modifying and merging images from Servier Medical Art,^26^ part of Laboratoires Servier, licensed under a Creative Commons Attribution 3.0 Unported License.

A schematic of the measurement protocol and probe positioning is provided in Fig. 1. One optical probe was placed on the forehead of the recruited subject while at the bedside (cerebral placement). CT scans were checked prior to the measurements whenever available to inspect the tissue and decide the optimal positioning. Accordingly, the hemisphere more easily accessible and/or without underlying trauma was always preferred for the probe placement. A second probe was preferably placed on the quadriceps femoris or, if not accessible, on the brachioradialis muscle (peripheral placement).

After the positioning of the optical probes, critical care personnel re-verified the blood bag compatibility with the patient, and the standard transfusion procedure was followed.

The continuous monitoring of the patients was carried out before, during, and after transfusion with a protocol of 30 min of baseline (pre-RBCT), 2- to 3-h-long RBCT, and 30 min of observational post-transfusion monitoring (post-RBCT). The timing of each period was adjusted according to the situation, as illustrated in Fig. 1.

Standard vials of blood samples were extracted from an artery (generally the arteria radialis) through a catheter during the protocol at baseline when half the blood bag was transfused, and during the observational final period, for a total of three times. In the ideal scenario, the initial and final blood samples were taken ∼10  min after each protocol part began but were adjusted according to the decisions of the medical personnel.

To standardize the protocol and its duration, we have decided to carry out our study only after one single RBCT even in those cases where more blood bags were indicated to restore the patient’s hemoglobin values.

### 2.3 Clinical Monitoring

Standard bedside monitors were used to collect physiological and clinical parameters. Mean arterial blood pressure and heart rate were measured by available sensors and were recorded by the critical care monitor (IntelliVue MX800 or MX750, Koninklijke Philips N.V., Amsterdam, Netherlands). Peripheral arterial oxygen saturation was measured by a standard pulse-oximeter (Nellcor DS100A-1, Medtronic, Minneapolis, Minnesota, United States) through a capnograph with a pulse-oximeter unit (Capnostream™ 20p, Medtronic).

A dedicated software (LabChart, version 6 and 8.1, ADInstruments Ltd, Oxford, United Kingdom) and hardware (PowerLab, ADInstruments Ltd) recorded all digitized vital signals at a sampling frequency of 400 Hz alongside a synchronization signal from the hybrid DO device.

The samples of arterial blood were analyzed by a co-oximeter (GEM Premier 4000, Werfen, Spain) to obtain the levels of arterial carbon dioxide (${\mathrm{PaCO}}_{2}$), ${\mathrm{HgbO}}_{2}$, arterial reduced hemoglobin concentration (Hgbr), arterial total hemoglobin concentration (Hgb), and HCT.

Patient demographic information was also collected, including sex, age, and etiology.

### 2.4 Optical Monitoring

A hybrid DO device, previously described in Refs. 27 and 28 and similar to Ref. 29 comprising of diffuse correlation spectroscopy (DCS) and time-resolved spectroscopy (TRS), was used for the non-invasive optical measurements. Both techniques are based on diffuse near-infrared spectroscopy methods, which exploit the safe interaction of low-powered laser sources (i.e. 3 to 27 mW) to investigate diffuse tissues, such as the human head.^30^ This non-invasive approach offers several advantages such as safety (light penetrates biological tissues relatively deeply with minimal health risks), patient comfort (is painless and is well tolerated by patients with respect to invasive transducers), and continuous monitoring of physiological changes during the therapy administration.

DCS quantifies the motion of scatterers inside of the probed tissue volume, primarily red blood cells, which was demonstrated to be related to the microvascular BF by analyzing the fluctuations in the intensity of the scattered light and obtained through a fitting process.^31^ TRS recovers the wavelength-dependent absorption and scattering coefficients, inherent optical properties of the probed tissue volume.^30^^,^^32^ In human tissues, these quantities allow one to retrieve ${[\mathrm{HbO}}_{2}]$, [Hhb], [HbT], and ultimately, ${\mathrm{StO}}_{2}$.^30^^,^^32^ Further details about the optical device, probe adhesion, and data acquisition can be found in Ref. 27. By combining DCS and TRS, a more comprehensive picture of tissue physiology can be obtained. Technical details about how this was achieved, especially at the probe level, can be found in Ref. 33, ensuring similar probed volume for both techniques. Briefly, the source-detector distance for TRS was 30 mm whereas 25 mm for the DCS. To minimize the crosstalk between the two methods, sources and detectors were placed on a line and intertwined as it follows: TRS source, DCS source, TRS detector, and DCS detector. DCS used a 785-nm continuous-wave laser source, which was filtered on the TRS detection side with a custom clean-up filter centered on 785 nm. 690- and 830-nm wavelengths were used for TRS. TRS-derived optical properties were fed to the DCS model for the retrieval of the BF for a more accurate solution while averaging the reduced scattering coefficient from different TRS wavelengths, as described in Ref. 33.

Real-time movement monitoring was employed using accelerometers on programmable boards placed at the two probe locations. This allowed for the monitoring and removal of potential artifacts due to motion.

Events were marked within the software routine, and data were synchronized with high precision with the ones provided by the clinical monitors, as described above.

### 2.5 Data Evaluation

DO analysis for the collected data followed the standard procedures for fitting methods for both TRS and DCS techniques^30^^,^^32^^,^^34^ and making use of Matlab (Release 2018b, The MathWorks, Inc. Natick, Massachusetts, United States). The optically derived parameters of interest in this study were: ${[\mathrm{HbO}}_{2}]$, [Hhb], [HbT], BF, OEF, and ${\mathrm{MRO}}_{2}$. Again, each acronym is preceded by a “C” when referred to the cerebral tissue and by a “P” when peripheral tissue. Probe placement was described above and shown in Fig. 1.

Strict rejection criteria were applied to the data according to the signal-to-noise ratio (SNR) and the measurement session quality, as detailed in Ref. 27. In addition, accelerometer information was visually inspected when an artifact was suspected from the marks to qualitatively identify motion artifacts. In this work, the SNR for TRS was defined as the ratio between the maximum of the TRS distribution of time-of-flight, which is the retrieved curve built by the photon distribution after the initial laser pulse traverses the tissue, and the standard deviation of a background portion of the same signal prior to the curve. The threshold value was SNR>10. DCS measurements were rejected if the averaged count rate of the detector channels to build the autocorrelation function was lower than 10 kHz and the β parameter was lower than 0.4.^30^

The non-rejected time traces of the mentioned variables were then synchronized with the clinical signals by exploiting the common time-base signal. Further parameters, such as OEF and ${\mathrm{MRO}}_{2}$, based on both optical and clinical data, were derived, as described in Refs. 27, 30, and 34.

As recent studies showed the need to apply corrections to DO signals calculations when HCT changes significantly during measurements,^35^^,^^36^ a correction in the ${\mathrm{MRO}}_{2}$ time-traces calculation was implemented. Accordingly, ${\mathrm{MRO}}_{2}$ calculation was also corrected by the HCT value measured by the blood gas analysis and similar to Ref. 37. To do so, in the following expression

$${\mathrm{MRO}}_{2}=\mathrm{BFI}\times \mathrm{OEF}\times {\mathrm{CaO}}_{2},$$ (1)

the arterial oxygen content (${\mathrm{CaO}}_{2}$) was computed as

$${\mathrm{CaO}}_{2}=k\times \mathrm{Hgb}\times {\mathrm{SaO}}_{2},$$ (2)

where k was assumed equal to $1.36\;{\mathrm{mLO}}_{2}/1\mathrm{gHgb}$ for mammalians and the arterial total hemoglobin concentration was expressed as

$$\mathrm{Hgb}=\frac{\mathrm{HCT}\;(\%)}{100}\times \frac{\mathrm{MCHC}}{10}.$$ (3)

In the latter equation, MCHC is the mean corpuscular hemoglobin concentration and was assumed to be the average value 340 g/L. For ${\mathrm{SaO}}_{2}$, the time-trace collected by the additional monitors was used. As in this study, HCT was available in three moments only, the computation of ${\mathrm{MRO}}_{2}$ was done separately for each period of the protocol with its respective HCT value kept as a constant.

All the optically derived hemodynamic signals were calculated for cerebral and peripheral areas below the probe placements separately. PBF, POEF, ${\mathrm{PMRO}}_{2}$, and their cerebral counterpart time-traces were referred to their pre-RBCT baseline period by dividing the entire time-trace by the pre-RBCT mean value. The baseline pre-RBCT lasted from the beginning of the recording to the beginning of the RBCT, marked as “transfusion start.” In this case, an “*r*” was added to describe these variables as relative (i.e., relative CBF = rCBF). Instead, [HbT], ${[\mathrm{HbO}}_{2}]$, and [Hhb] were calculated as difference changes, and therefore, each time trace was subtracted by its average over the baseline pre-RBCT period. This led to Δ[HbT], Δ[Hhb], and $\Delta {[\mathrm{HbO}}_{2}]$ for both peripheral and cerebral probes.

The data related to vital signals collected by external available monitors were read in software during post-processing and synchronized with the optical time traces. All data were down-sampled to 10-s bins.

Marked events were reported on all physiological time-traces. This led to further artifact identification and removal corresponding to periods of patient manipulation due to clinical procedures. Similarly, the visual inspection of data allowed the identification of features such as movement artifacts.

Relevant blood-derived parameters obtained by the output of the blood gas analyzer were collected for each subject in a database together with all demographic and clinical patient information.

### 2.6 Statistical Analysis

All statistical analyses were carried out with R (v4.0.1, R Core Team, Auckland, New Zealand, United States) and the integrated development environment RStudio (v1.1.5042, RStudio, Inc., Boston, Massachusetts, United States). In particular, the nlme v. 3.1-152, lme4 v. 1.1-27, lmerTest v. 3.1-3, and stats v. 3.6.3 packages were utilized.

Descriptive statistics were computed for each variable. Clinical variables and demographic information (i.e., sex) were summarized as mean[standard deviation (SD)] or proportions (i.e., males:females as M:F).

Variables obtained at specific time points by blood gas samples (up to three times per measurement session), that is, Hgb, HCT, and ${\mathrm{PaCO}}_{2}$, were summarized by mean (SD) both before (pre-) and after (post-) transfusion. Pre-transfusion values were compared with post-transfusion ones by Wilcoxon paired tests.

Continuous normally distributed optically derived hemodynamic time traces underwent a windowing procedure to define pre- and post-transfusion values, both at the cerebral and peripheral levels. The pre-transfusion window was selected lasting the entire pre-RBCT period, prior to marking the beginning of the RBCT. The post-transfusion window included 6 min after the marked event of RBCT end. The values within the windows were averaged and collected separately into two distributions, one for the cerebral and one for the peripheral placement.

For each variable, the difference between the post-transfusion distribution of mean values with respect to its pre-RBCT distribution was tested by unpaired Wilcoxon test: the mean of the pre-RBCT was subtracted to the post-RBCT distribution (providing a relative mean) and its difference from zero tested. For the distributions of variables, which were expected to increase after transfusion, one-sided Wilcoxon tests were used to check whether the relative change was greater than 0 (with “greater” option for the test applied), whereas one-sided test was used to check whether the relative mean was less than 0 (with “less” option employed) for those variables expected to show a decrease. Significance was attributed when p-values (*p*) were lower than 0.05, and all values lower than 0.001 were reported only as p<0.001.

Furthermore, the paired Wilcoxon test was used to compare cerebral and peripheral pre-transfusion distributions of values to make sure that there were no initial differences (thus discarding cases where one placement was not available, that is, cerebral missing but peripheral available). Paired Wilcoxon tests were also used to test the difference between cerebral and peripheral data distributions post-transfusion.

Boxplots [median, first, and third interquartile ranges (IQR)] for all pre- and post-RBCT distributions of mean values and variables, separated between cerebral and peripheral placements, were depicted to visualize the data. The median values with respect to the baseline pre-RBCT were used to identify the direction of the change for the distribution, for instance, an increase if it was greater than the baseline and statistically significant.

## 3 Results

### 3.1 Population Characteristics

Fifteen subjects were recruited (see Table 1) with 10 patients with polytrauma, three with ischemic or hemorrhagic stroke, and two with severe burns. The data from one subject had to be discarded due to poor SNR in the optical measurement (according to the criteria described above) both on the head (effect of the trauma) and the peripheral muscle (scar tissue due to an old burn). Furthermore, two data sets from the probe in the cerebral placement were discarded due to poor SNR.

**Table 1:** Demographic and clinical data. Demographic and clinical data (n=15).^a^ Patient ID = 3 was discarded.

ID	Sex	Age	Polytrauma	TBI	GCS	Type of cerebral lesion^a^	Etiology	Hgb pre (g/dL)	Hgb post (g/dL)	HCT pre (%)	HCT post (%)	RBCs storage (days)
1	F	21	Yes	Yes	4	DI-IV	Traffic accident (motorcycle)	8.5	9.6	26	29	30
2	M	18	Yes	Yes	5	EML1	Traffic accident (car)	NA	NA	NA	NA	16
3	M	55	No	No	15	Not applicable	Suicide attempt (severe burns)	7.6	8.9	23	27	21
4	M	44	Yes	Yes	14	DI-I	Traffic accident (motorcycle)	8.2	8.9	25	27	16
5	M	16	Yes	Yes	7	DI-II	Traffic accident (motorcycle)	8	9.2	24	28	17
6	M	24	Yes	Yes	15	DI-I	Traffic accident (motorcycle)	8.2	8.9	25	27	26
7	M	70	No	No	15	Not applicable	Stroke	9.2	9.8	28	29	29
8	F	72	Yes	Yes	15	DI-I	Fall from height >3 m	7.6	8.7	23	26	25
9	M	22	Yes	Yes	11	EML2	Fall from height >3 m	8.9	10.5	27	32	21
10	M	50	Yes	Yes	14	DI-I	Traffic accident (motorcycle)	7.8	8.9	23	27	25
11	F	65	No	No	15	Not applicable	Stroke	7.9	9	24	27	21
12	M	51	No	No	15	Not applicable	Cerebral hematoma	7.8	8.8	23	26	21
13	M	27	Yes	Yes	6	DI-I	Hit by a train	7.9	8.7	24	26	20
14	M	44	Yes	No	15	Not applicable	Traffic accident (motorcycle)	7.6	8.4	23	25	17
15	M	26	No	No	15	Not applicable	Severe burns	8	9.1	24	27	12

a Type of cerebral lesion according to the Traumatic Data bank classification^38^ in the computed tomography scan closest to the study. Diffuse injury I (DI-I): no visible intracranial patholoseen on CT scan; diffuse injury II (DI-II): cisterns are present with midline shift 0-5 mm and/or high or mixed-density lesions ≤ 25cc; diffuse injury III (DI-III): cisterns compressed or absent with midline shift 0-5 mm and/or high or mixed-density lesions ≤ 25cc; diffuse injury IV (DI-IV): midline shift >5mm without high or mixed-density lesion > 25cc; evacuated mass lesion (EML): any lesion surgically evacuated; non-evacuated mass lesion (NEML): high or mixed-density lesion > 25cc not surgically evacuated. 1: Epidural hematoma. 2: Subdural hematoma.

F: Female; GCS: Glasgow Coma Scale score, value at hospital admission; Hgb pre: blood hemoglobin concentration before transfusion (g/dL); Hgb post: blood hemoglobin concentration after transfusion (g/dL); M: Male; RBCs: Red blood cells; TBI: traumatic brain injury; NA: not available.

Consequently, 14 subjects were included in the following analysis out of which all the measurement sessions had peripheral measurements of the optical variables (time traces), whereas 12 also had cerebral hemodynamic time traces. Moreover, in one subject, the peripheral measurement had to be carried out on the brachioradialis muscle due to the inaccessibility of the quadriceps femoris muscle.

In one subject, a vial of blood sample was extracted only prior to the measurement due to the care provider’s decision. In this case, for the hematocrit correction of ${\mathrm{MRO}}_{2}$, it was decided to use as the post-transfusion value, the average over all of the other subjects’ post-transfusion HCT values, which had started from the same initial HCT value as this subject.

The mean age of the 14 included patients was 39(19) years (median: 35, minimum: 16, maximum: 72 years), and the proportion of males to females was 11:3. Table 1 summarizes the demographic and clinical data of the patients. Descriptors for the variables related to the blood gas samples are summarized in Table 2. For the included subjects, the average RBC bag volume was 306(22) mL, and the average bag age was 21(5) days.

**Table 2:** Pre- and post-transfusion values by blood gas analysis. Results in terms of mean(SD) for Hgb, ${\mathrm{PaCO}}_{2}$ and HCT values obtained by blood gas analysis pre- and post-transfusion (pre- RBCT and post-RBCT). Post-transfusion values were tested against pre-transfusion values to verify whether had undergone a statistically significant change [significant when the *p*-value (*p*) < 0.05]. In addition, an upward arrow indicates that the post-RBCT values increased (the median value for the distribution was higher than for the pre-RBCT values), whereas a downward arrow stays for a decrease.

Variable	Pre-RBCT	Post-RBCT	Dir.	*p*-value
Hgb (g/dL)	8.1(0.48)	9.1(0.55)	↑	p<0.001
${\mathrm{PaCO}}_{2}$ (mmHg)	39.4(4.3)	40(4.5)	—	p=0.22
HCT (%)	24.5(1.6)	27.4(1.8)	↑	p<0.001

Hgb, arterial total hemoglobin concentration, measured by blood gas analysis; ${\mathrm{PaCO}}_{2}$, arterial pressure of carbon dioxide; HCT, hematocrit; RBCT, red blood cell transfusion; Dir., direction of change.

### 3.2 Representative Data During Transfusion

**Fig. 2:**
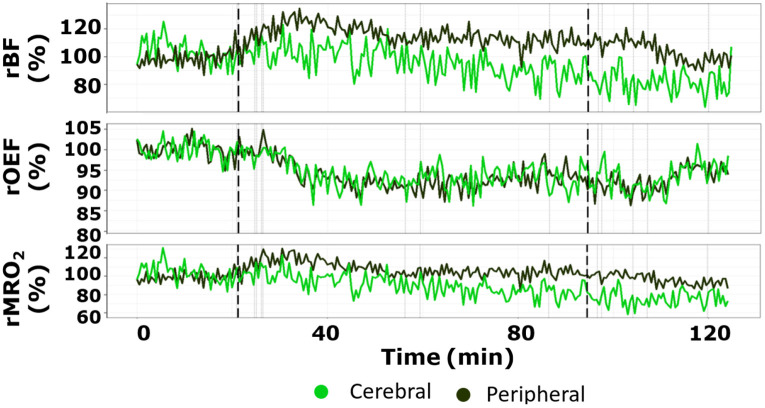
Time-traces example. Time traces collected during the RBCT of a patient for an entire protocol, comprising baseline pre-RBCT, blood unit transfusion, and post-transfusion period. The signals are depicted with peripheral and cerebral recordings color-coded, as shown in the legend. The beginning and end of the process are highlighted by thick dashed vertical lines, synchronized in time for all variables. Additional events marked during the protocol are represented by vertical lines.

A representative set of data (male, 18 y.o. with polytrauma and severe TBI) is presented in Fig. 2 (case 2 in Table 1) where darker curves show data from the peripheral muscle (rPBF, rPOEF, ${\mathrm{rPMRO}}_{2}$) and the lighter curves correspond to data from the brain (rCBF, rCOEF, ${\mathrm{rCMRO}}_{2}$). The start and the end of the RBCT are indicated by vertical dashed lines. Additional events, such as blood gas sampling and movements, are depicted by lighter vertical lines. Initial Hgb, HCT, and ${\mathrm{PaCO}}_{2}$ values were 7.9 g/dL, 26%, and 36 mmHg, respectively.

By eye, it can be seen that there is a general trend for the rCBF to decrease with respect to the pre-RBCT period, whereas the rPBF has an increasing trend. Instead, rOEF shows a decreasing trend for both peripheral and cerebral signals. The response of the metabolic rate of oxygen extraction is more subtle with a small decrease in ${\mathrm{rCMRO}}_{2}$ by the end of the protocol.

For completeness, the time traces of ${\mathrm{rMRO}}_{2}$ for all subjects are provided in Appendix B.

### 3.3 Pre- and Post-Transfusion Comparisons

As far as blood gas sample-related variables are concerned, the results of the comparison between pre-RBCT and post-RBCT distributions of mean values to check whether a significant change occurred are provided in Table 2, where the *p*-values and direction of changes are reported. RBCT caused a significant increase in the Hgb by ∼0.9  g/dL (p<0.001) and HCT measured by blood gas samples by ∼3% (p<0.001). ${\mathrm{PaCO}}_{2}$ did not show any significant change (p=0.22).

**Fig. 3:**
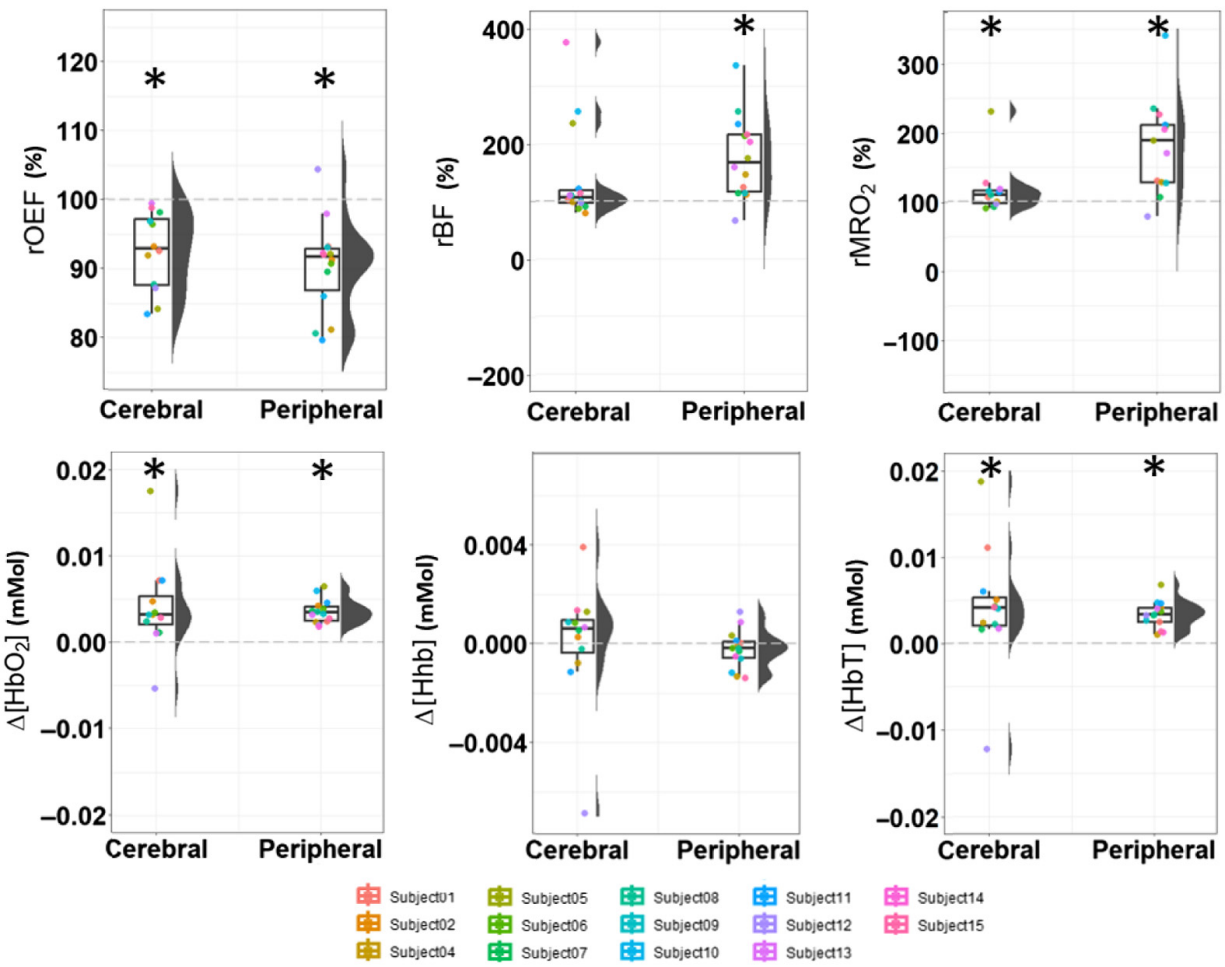
Group distributions relative to baseline. Graphs representing the post-transfusion data distribution for all optically derived variables and divided into cerebral and peripheral muscle. For each distribution of mean values, its boxplot and violin plot are represented (median, first, and third quartiles). Dashed grey lines are drawn, indicating the baseline pre-RBCT values for the distributions, which they are relative to and compared with. Subjects are color-coded in the same way for all variables. An asterisk on the top of a boxplot indicates that the distribution tested significantly different than the baseline. No significant difference was found between peripheral and cerebral post-transfusion values apart from the metabolic rate of oxygen. rOEF, relative oxygen extraction fraction; rBF, relative blood flow; ${\mathrm{rMRO}}_{2}$, relative metabolic rate of oxygen; $\Delta {[\mathrm{HbO}}_{2}]$, change in microvascular oxy-hemoglobin concentration; Δ[Hhb], change in microvascular deoxy-hemoglobin concentration; Δ[HbT], change in microvascular total hemoglobin concentration.

In relation to the tests for pre- and post-transfusion distributions from the hemodynamic time traces, the results are detailed in Table 3 and depicted in Fig. 3. Differences between cerebral baseline pre-RBCT distributions and peripheral baseline pre-RBCT distributions for all variables were not statistically significant (p>0.05) and were omitted from Table 3. All post-transfusion test results are provided together with the direction (Dir.) of the change with respect to the baseline. Moreover, the results of the tests between the post-RBCT cerebral values and the peripheral values are reported in the third column.

**Table 3:** Results of the optically derived variables. Summary of the results of all tests carried out for the optically derived variables (rBF, rOEF, ${\mathrm{rMRO}}_{2}$, $\Delta {[\mathrm{HbO}}_{2}]$, Δ[Hhb], Δ[HbT]). Under the column “cerebral,” the post-transfusion values were tested for difference with respect to baseline. The results concerning the post-transfusion muscle-related values are reported below “peripheral,” again tested with respect to baseline. Post-transfusion values for the cerebral level were tested for significant differences with respect to the post-transfusion values at the peripheral level in the last column (“cerebral versus peripheral”). Significance was defined as *p*-values lower than 0.05. The direction (Dir.) of the change of the median value for the distribution is represented by an arrow: upward if it increased and downward if decreased.

Variable	Cerebral		Peripheral		Cerebral versus peripheral	
	*p*-value	Dir.	*p*-value	Dir.	*p*-value	Dir.
rBF	0.07	—	<0.001	↑	0.1	—
rOEF	<0.001	↓	<0.001	↓	0.2	—
${\mathrm{rMRO}}_{2}$	0.03	↑	<0.001	↑	0.03	↑ ^a^
$\Delta {[\mathrm{HbO}}_{2}]$	0.008	↑	<0.001	↑	0.9	—
Δ[Hhb]	0.2	—	0.1	—	0.2	—
Δ[HbT]	0.01	↑	<0.001	↑	0.5	—

a In this case, the arrow indicates that the peripheral level was significantly higher than the cerebral level after RBCT.

rBF, relative blood flow; rOEF, relative oxygen extraction fraction; ${\mathrm{rMRO}}_{2}$, relative metabolic rate of oxygen; Δ[HbT], change in [HbT]; $\Delta {[\mathrm{HbO}}_{2}]$, change in ${[\mathrm{HbO}}_{2}]$; Δ[Hhb], change in [Hhb].

RBCT provoked a significant increase (p<0.001) in rPBF by 68% (by comparing the percent change between the median post- to median pre-values), whereas no significant change in rCBF was observed (p=0.07). The rPOEF median value fell significantly by 8% (p<0.001) and rCOEF by 7% (p<0.001) with respect to their pre-RBCT values. The median ${\mathrm{rPMRO}}_{2}$ significantly raised by 89% (p<0.001) and ${\mathrm{rCMRO}}_{2}$ by 10% (p=0.03), with a significantly greater median increase in the muscle by 79% than in the brain (p=0.03). No significant differences were found in the post-RBCT comparison for the other hemodynamic signals (p=0.1 for rBF, p=0.2 for rOEF).

Both $\Delta [{\mathrm{HbO}}_{2}]$ and Δ[HbT] had a significant increase both at cerebral and peripheral levels, with similar median shift with respect to baseline pre-RBCT values (as visible in Fig. 3). $\Delta [{\mathrm{HbO}}_{2}]$ increased by 0.0040(0.0053) mMol on the brain and 0.0036(0.0014) mMol on the muscle; Δ[HbT] raised by 0.0041(0.0071) mMol on the brain and 0.0034(0.0016) mMol on the muscle. Instead, Δ[Hhb] had no significant changes (p=0.2 and p=0.1 for cerebral and peripheral placements, respectively). No statistical difference was found between post-RBCT cerebral versus peripheral placement for any of the hemoglobin concentration-related variables.

## 4 Discussion

The study aimed to demonstrate the usefulness of hybrid DO to quantify the physiological changes after RBCT and to provide a preliminary characterization of the response at both the peripheral muscle and the brain in a small, pilot population of critically ill patients. We have obtained a rich array of signals. As the RBCT response for the invasive and standard variables is well documented, we focus on discussing the results of the optical monitoring with respect to the literature, contributing to a thorough characterization.

With respect to our objectives, we have shown that hybrid DO could be validated in the ICU scenario, monitoring critically ill patients undergoing RBCT. Moreover, we were able to quantify and evaluate changes in the measured parameters in correspondence with RBCT (by which we mean “happening simultaneously to,” in relation to cause–effect causality), similarly to the other non-optical parameters.

RBCT provoked a significant increase in rPBF, whereas no significant change in rCBF was observed. However, this is in contrast to the results obtained on the brain in ill but young populations (i.e., sickle cell disease, very preterm infants) where a decrease in the CBF was found after the intake of blood.^13^^,^^18^^,^^39^ For instance, in Ref. 18, the decrease of cerebral blood volume (CBV) was 0.5 mL/100g, whereas, in Ref. 39, CBV decreased by a median of 18.2% and CBF by a median of 21.2%. However, we acknowledge that the populations, the age, the origin of their anemia, and the etiology are not fully relatable and may account for these discrepancies.

As for alternative monitoring methods, for example, a reduction by 18% in the blood flow velocity measured by transcranial Doppler ultrasound was found in stroke patients after 3 h post-RBCT both on infarcted and non-infarcted regions, with significantly lower baseline values for the infarcted areas.^40^ Nonetheless, in another study involving subarachnoid hemorrhage (SAH) patients, post-RBCT global CBF values monitored by O15-positron emission tomography (PET), remained unchanged,^41^ similar to our findings. Differences in the post-RBCT CBF response between children and adults with sickle cell anemia were also found by magnetic resonance imaging (MRI) and magnetic resonance angiography:^42^ post-RBCT CBF did not significantly change in the adults (N=16), whereas it significantly decreased in the children (N=10, with −22.3  mL/100g/min in 9/10 participants). In Ref. 43, CBF evaluated in children with sickle cell anemia by MRI with arterial spin labeling significantly decreased by 6.5 mL/100g. These findings are mostly in contrast with our results. However, a possible explanation may lie again in the etiology itself and the age of the participants. Moreover, the differences may be due to the fact that we have decided to monitor only the transfusion of a single RBC bag, the first one, even when more units had been assigned to the patient. This was done to be consistent among patients and to compare the amount of change in the physiological signals. According to the literature, greater changes are expected when more bags are transfused.^44^

Another set of parameters that we report is related to blood oxygenation and total hemoglobin concentration. Here, we have used TRS, which is well-known to be more accurate and precise compared with the commercially and clinically available continuous-wave NIRS, which dominates the literature. However, for the purposes of this pilot study, we do not get into a discussion of these differences and discuss the findings together. Moreover, as several NIRS studies characterized the tissue oxygen saturation (${\mathrm{StO}}_{2}$) rather than OEF, which is generally inversely related to the OEF, we will use it as well as an inverse comparison to our findings. In our study, at the cerebral level, we have encountered a significant decrease in rCOEF, a significant increase in $\Delta [{\mathrm{HbO}}_{2}]$ and Δ[HbT], and no change in Δ[Hhb]. For rCOEF, this is in agreement with the literature and was mainly confirmed in young populations after RBCT;^5^^,^^13^^,^^17^^,^^45^ although not always, a significant change was found.^24^ A significant decrease of rCOEF by 0.06% by CW-NIRS was reported, for instance.

Studies using other modalities reported a significant decrease in post-RBCT rCOEF, which was observed both in children and adults with sickle cell anemia evaluated by MRI (−0.1),^42^ in children with sickle cell anemia assessed by asymmetric spin echo sequence MRI technique (−2% for the whole brain)^43^ and in SAH patients by PET (−0.11).^41^ These results are consistent with ours. Our results are also strengthened by several studies highlighting a significant increase in cerebral ${\mathrm{StO}}_{2}$.^15^^–^^19^^,^^21^^,^^23^^,^^46^^,^^47^ In Ref. 15, cerebral regional ${\mathrm{StO}}_{2}$ measured by CW-NIRS increased by 17% in infants and by 4.2% in patients undergoing transfusion during aortic or spinal surgery.^21^ Only in Ref. 24, McCredie et al. did not detect any significant change in cerebral ${\mathrm{StO}}_{2}$ by bifrontal NIRS measurements in TBI after RBCT. Only one study used a TRS device in infants^18^ and found the same: cerebral ${\mathrm{StO}}_{2}$ increased by ∼2% after transfusion. As for $\Delta [{\mathrm{HbO}}_{2}]$ and Δ[HbT], our findings are in line with other studies using NIRS.^14^^,^^18^^,^^24^^,^^46^ For instance, in Ref. 14, $\Delta [{\mathrm{HbO}}_{2}]$ was 0.026 mMol, and Δ[HbT] was 0.02 mMol, which was in the same direction as our study, although with greater absolute value and measured by a frequency domain tissue oximeter.

In our study, at the peripheral level, we have found a significant decrease in rPOEF, a significant increase in $\Delta [{\mathrm{HbO}}_{2}]$ and Δ[HbT], but no significant change in Δ[Hhb]. The rPOEF result is in agreement with the literature,^5^^,^^13^^,^^17^^,^^45^ where a 0.13% significant decrease in the abdomen was found, for example, comparable to ours. Conversely, several studies highlighted a significant increase in the ${\mathrm{StO}}_{2}$ peripheral level,^15^^–^^19^^,^^21^^,^^23^^,^^46^^,^^47^ which is also in accordance with our study. For instance, peripheral ${\mathrm{StO}}_{2}$ increased by 17% in infants^15^ and by 1.6% in patients undergoing transfusion during aortic or spinal surgery.^21^ The peripheral increase in $\Delta [{\mathrm{HbO}}_{2}]$ and Δ[HbT] was confirmed by NIRS in the literature.^14^^,^^18^^,^^24^^,^^46^ Regarding Δ[Hhb], a similar result was found interscapularly by CW-NIRS.^14^ However, Δ[Hhb] was often reported to decrease in the literature.

It is possible to speculate that the variations in [Hhb] were too small and could have probably been more relevant in the case of full restoration to normal hemoglobin concentration and hematocrit values, which were not reached by the end of the measurements due to our protocol choice. Furthermore, some studies highlighted RBCT failure in raising the oxygen uptake except that there is a dependency between $O_{2}$ uptake and ${\mathrm{DO}}_{2}$, which is generally true only in severe anemic conditions. Generally, more pronounced changes in the brain were found for groups with lower hemoglobin (<9.7  g/dL).^5^^,^^45^

Both ${\mathrm{rPMRO}}_{2}$ and ${\mathrm{rCMRO}}_{2}$ raised due to RBCT, with a greater median increase in the muscle than in the brain and with a statistically significant difference between the two. This result is in disagreement with what was found in some literature related to infants where, for instance, a decrease^13^ or no change (p>0.05)^39^^,^^48^ had been found in ${\mathrm{CMRO}}_{2}$ by optical methods. Likewise, no ${\mathrm{rCMRO}}_{2}$ change was found in SAH patients by PET^41^ nor in children with sickle cell anemia by MRI.^43^ Further research is needed to clarify these discrepancies.

As for the difference between cerebral and peripheral level response after RBCT, significant statistical difference was confirmed only for ${\mathrm{rMRO}}_{2}$ tests.

Overall, the findings for the cohort highlight an improvement in oxy- and total hemoglobin concentration and the oxygen supply, both by optical and standard methods, as expected after RBCT. Moreover, with the obtained information, it is possible to evaluate on an individual basis whether these changes pose any risks or lack them. Indeed, although anemia and RBCT are crucial for a patient’s recovery, collateral issues may arise, especially in cases of impaired cerebral autoregulation, potentially leading to stemming misery perfusion with consequent risk of cerebral ischemia. In light of the post-RBCT findings for the cohort, for instance, we did not identify any misery perfusion, as defined in Ref. 27, nor a hyperperfusion or hypoperfusion, because the rCBF did not significantly change and the rCOEF significantly decreased.

Cerebral autoregulation is often impaired in critically ill subjects,^49^^,^^50^ especially in TBI.^51^ In such eventuality, systemic changes may directly affect the brain and damage it.^51^^,^^52^ Therefore, it is reasonable to assume that if a robust monitor of cerebral autoregulation becomes available, it would be recommendable to utilize it frequently. In our study, the significant decrease in rCOEF along with no change in the rCBF and ${\mathrm{rCMRO}}_{2}$ suggests that the cerebral autoregulation was at play. In fact, the decrease in the rCOEF indicates that the brain was extracting less oxygen from the blood, potentially allowing for better ${\mathrm{DO}}_{2}$ to meet the metabolic demand. This is further supported by the lack of change in rCBF and ${\mathrm{rCMRO}}_{2}$, indicating a regulation mechanism to maintain consistent oxygen supply. Conversely, the significant rPBF and ${\mathrm{rPMRO}}_{2}$ increase in quadriceps along with decreased rPOEF indicates hyperemia or luxury perfusion at the peripheral level. This suggests an increased supply of oxygen to the quadriceps muscle, possibly to meet increased metabolic demands, that does not imply deleterious consequences. These are rather adaptive responses to ensure adequate oxygen supply and brain protection.

The first strong limitation of this study is the small sample size. This study was a good starting point to prove the feasibility of this method and spare the need for larger clinical trials.

Once more, we recognize that our study protocol design based only on the monitoring during the first RBC bag transfusion may have been a limitation in the sense of the lower response associated with it than multiple bags. In fact, greater blood exchange may have led to potentially greater changes in our variables and could be worth further exploration to verify whether the results keep consistent over several bags or lead to significant changes.

Furthermore, we acknowledge that the heterogeneity of the patient’s condition prior to RBCT should be addressed and taken into consideration as it plays an important role in the clinician’s decision-making upon RBCT. However, this would only be possible with a larger dataset.

Another important limitation is the fact that, as mentioned in Sec. 2.5, the correction for the hematocrit value was taken into account in the calculation of the ${\mathrm{MRO}}_{2}$ in the analysis. However, in Ref. 36, a correction method was outlined for the BFI in phantom experiments. This approach is currently under validation against MRI measurements^39^ by the same group, and if confirmed *in vivo*, it would be a further step to implement in the future. Moreover, another approach was very recently outlined in Ref. 53. Therefore, our calculations of the BFI should be considered preliminary.

Further limitation can be considered the placement of the probe on a different muscle for one subject which may introduce some variability due to potential differences in hemodynamic and metabolic responses, we believe that the limited sample size and the nature of the study minimize the impact of this variation on our overall conclusions. To reduce potential confounding effects, future studies should maintain consistent probe placement and/or utilize the muscle type/location as a confounding variable in statistical analysis.

The limited number of female participants is another limitation of our study. However, due to the specific nature of our study and the limitations of our sample size, a detailed analysis of sex differences was not statistically feasible. This study did not take any specific action to select patients based on biological sex, and all eligible subjects were approached for recruitment. Similar considerations apply to race/ethnicity, which were not considered here. We acknowledge the importance of further research in a larger clinical trial in the future.

Despite RBCT’s role in critical care (improving oxygen delivery), optimal hemoglobin thresholds for transfusion in critically ill patients, especially TBI, remain debated.^2^^,^^4^^–^^6^^,^^8^^–^^10^ It is the clinicians who carefully weigh risks and benefits to decide on RBCT, considering factors such as illness severity, blood pressure stability, brain function, and underlying health conditions. The clinician also chooses the appropriate blood product, volume, and transfusion rate considering the patient’s blood type, specific needs, and risk of reactions. They then monitor the patient’s response to assess transfusion effectiveness. Recent concerns highlight potential negative effects of RBCT in TBI due to increased blood viscosity and impaired cerebral autoregulation.^2^ This has led to a shift toward more restrictive transfusion strategies.^1^^–^^4^^,^^9^^,^^11^^,^^12^ Nonetheless, the optimal threshold is patient-specific. Several studies proposed moving beyond hemoglobin levels and using surrogate markers for oxygen delivery issues.^6^^,^^21^^,^^54^ NIRS-derived parameters, such as cerebral ${\mathrm{StO}}_{2}$, show promise as additional data points for transfusion decisions, potentially enabling personalized thresholds based on brain oxygen needs and contributing to the algorithm decision-making.^10^^,^^20^

Finally, it is essential to acknowledge the potential influence of the well-known partial volume effect in our study, which is a recognized limitation when using techniques such as DCS and TRS.^55^^,^^56^ In fact, they are susceptible to variations in tissue composition within the measurement volume, and the presence of different morphological tissue types and structures can lead to uncertainties in the interpretation of the acquired data, for instance, the underestimation of rCBF and rOEF. Although we made efforts to minimize this effect by carefully selecting measurement locations, the inherent challenge of disentangling signals from multiple tissue types, especially the extracerebral layer, was not entirely eliminated. More advanced analysis could be employed to do so. In our case, the probed tissue composition below the probes also additionally considerably varied between cerebral and peripheral measurement locations.

## 5 Conclusion

This work focused on establishing the compatibility of DO measurements and critically ill subjects, with responses to RBCT that are more unpredictable with respect to other populations, and quantified it both at cerebral and peripheral levels. However, the applicability could be extended to other populations where anemia is a trigger to RBCT, such as during some types of surgery.

We have shown the potential usefulness of hybrid DO in RBCT management, ideally aiming at improving RBCT decision-making in the future and paving the way to a widespread implication of hybrid DO monitors in clinical trials about RBCT. The acquired knowledge in this important clinical field could even help in personalizing RBC therapy in critical patients and enhance the quality of transfusion therapies and strategies, avoiding deleterious consequences such as hypoxia, hypoperfusion, hyperperfusion, ischemia, and transfusion-associated circulatory overload.

## 6 Appendix A: Terminology and Acronyms

In this section, we clarify the distinction between the parameters derived by blood gas sample analysis from the ones by DO measurements. In fact, these two methods/fields often use the same terminology to define similar parameters although of another origin.

The oxy-hemoglobin concentration that is calculated from a blood gas sample by a co-oximeter refers to the fraction of the amount of hemoglobin present in the arterial blood that is bound to the oxygen: it corresponds to the level of oxygenated hemoglobin in the blood, defining its capability to carry oxygen. In this article, we have used the acronym ${\mathrm{HgbO}}_{2}$ and Hgbr for the derived oxy- and reduced hemoglobin concentrations. As for the arterial total hemoglobin concentration, it includes the cumulative concentration of various forms of hemoglobin, such as carboxyhemoglobin and methemoglobin, in addition to the previously mentioned ones. We used Hgb to refer to it.

Oxy- and deoxy-hemoglobin concentrations that are derived from TRS measurements provide a non-invasive estimate of the content of such chromophores from deep tissue microvasculature, locally related to a specific tissue region and the underneath volume. This method does not derive these components from the blood only, rather from an average between all tissues regionally interacting with the light source and within its penetration depth. Among the usual acronyms used for optical hemoglobin concentrations, in this article, we have decided to adopt the following ones to avoid confusion: microvascular deoxy-hemoglobin concentration ([Hhb]) and microvascular oxy-hemoglobin concentration (${[\mathrm{HbO}}_{2}]$). The total microvascular hemoglobin concentration is calculated from the sum of these two latter and acronymized as [HbT].

We summarize this in Table 4.

**Table 4:** Acronyms of the parameters related to hemoglobin concentration to clarify which one is derived by which method.

Acronym	Parameter	Method
Hgb	Arterial total hemoglobin concentration	Blood gas analysis
${\mathrm{HgbO}}_{2}$	Arterial oxy-hemoglobin concentration	Blood gas analysis
Hgbr	Arterial reduced hemoglobin concentration	Blood gas analysis
[HbT]	Microvascular total hemoglobin concentration	Diffuse optics
${[\mathrm{HbO}}_{2}]$	Microvascular oxy-hemoglobin concentration	Diffuse optics
[Hhb]	Microvascular deoxy-hemoglobin concentration	Diffuse optics

## 7 Appendix B: rMRO_2_ Time-Traces

**Fig. 4:**
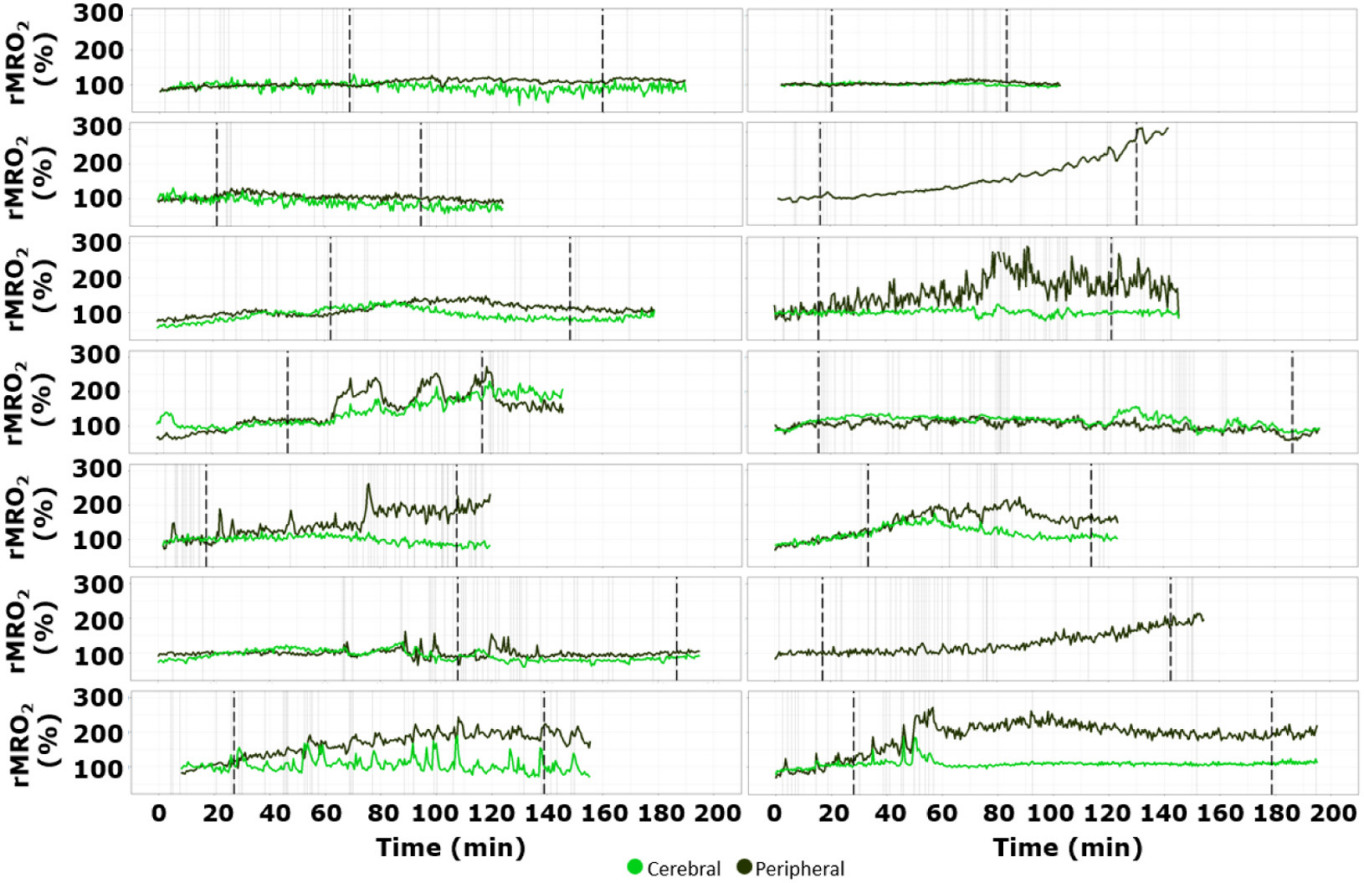
${\mathrm{rMRO}}_{2}$ time-traces for all subjects. ${\mathrm{rMRO}}_{2}$ time-traces for all subjects showing baseline pre-RBCT, blood unit transfusion and post-transfusion period. The beginning and end of the process are highlighted by thick dashed vertical lines, synchronized in time for all variables. Additional events marked during the protocol are represented by vertical lines. The signals are depicted with peripheral (dark green) and cerebral (light green) recordings color-coded as shown in the legend.

In this section, we showcase the ${\mathrm{rMRO}}_{2}$ time-traces for all subjects, including the one in Fig. 2, both at cerebral and peripheral level in Fig. 4, making it easier to compare them.

## Data Availability

Data are available in the Zenodo repository at DOI: 10.5281/zenodo.13208341.
